# Bone-Targeted Alkaline Phosphatase Treatment of Mandibular Bone and Teeth in Lethal Hypophosphatasia via an scAAV8 Vector

**DOI:** 10.1016/j.omtm.2018.08.004

**Published:** 2018-08-18

**Authors:** Ryo Ikeue, Aki Nakamura-Takahashi, Yuko Nitahara-Kasahara, Atsushi Watanabe, Takashi Muramatsu, Toru Sato, Takashi Okada

**Affiliations:** 1 Department of Fixed Prosthodontics, Tokyo Dental College, Tokyo, Japan; 2 Department of Pharmacology, Tokyo Dental College, Tokyo, Japan; 3 Department of Biochemistry and Molecular Biology, Nippon Medical School, Tokyo, Japan; 4 Division of Clinical Genetics, Nippon Medical School Hospital, Tokyo, Japan; 5 Department of Operative Dentistry, Tokyo Dental College, Tokyo, Japan

**Keywords:** hypophosphatasia, AAV vector, alkaline phosphatase, gene therapy, mandibular

## Abstract

Hypophosphatasia is an inherited disease caused by mutations in the gene encoding tissue-nonspecific alkaline phosphatase (TNALP), the major symptom of which is hypomineralization of the bones and teeth. We had recently demonstrated that TNALP-deficient (*Akp2*^*−/−*^) mice, which mimic the phenotype of the severe infantile form of hypophosphatasia, can be treated by intramuscular injection of a self-complementary (sc) type 8 recombinant adeno-associated virus (rAAV8) vector expressing bone-targeted TNALP with deca-aspartates at the C terminus (TNALP-D_10_) via the muscle creatine kinase (MCK) promoter. In this study, we focused on the efficacy of this scAAV8-MCK-TNALP-D_10_ treatment on the mandibular bone and teeth in neonatal *Akp2*^*−/−*^ mice. Upon scAAV8-MCK-TNALP-D_10_ injection, an improvement of mandibular growth was observed by X-ray analysis. Micro-computed tomography analysis revealed progressive mineralization of the molar root in the treated *Akp2*^*−/−*^ mice, and morphometric parameters of the alveolar bone were improved. These results suggest that the mandibular bones and teeth of hypophosphatasia were effectively treated by muscle directed rAAV-mediated TNALP-D_10_ transduction. Our strategy would be promising for future hypophosphatasia gene therapy because it induces dentoalveolar mineralization and reduces the risk of tooth exfoliation.

## Introduction

Hypophosphatasia (HPP) is an inherited disease caused by mutations in the gene encoding tissue-nonspecific alkaline phosphatase (TNALP), an enzyme that reduces local concentrations of the mineralization inhibitor inorganic pyrophosphate (PPi).^1^ The major symptom of HPP is hypomineralization of the bones, and other symptoms include respiratory dysfunction, epileptic seizures, and dental defects, including cementum deficiency, premature tooth exfoliation, thin dentin, widened pulp chambers, and enamel hypoplasia.^2^, ^3^, ^4^ HPP exhibits a wide range of clinical severity, from a lethal perinatal form to a mild odontodysplasia (odonto-HPP) that is characterized only by dental abnormalities.^5^, ^6^

Although an effective treatment of HPP was not established until recent years, enzyme replacement therapy (ERT) using bone-targeted TNALP with a deca-aspartate (D10) sequence at the C terminus (TNALP-D_10_) was commercially approved in Japan in 2015. The clinical benefits of ERT were acknowledged because TNALP is an ectoenzyme that attaches to the outer cell membrane via a glycosyl-phosphatidylinositol (GPI) anchor.^5^ TNALP-D_10_ is bioengineered with the C terminus extended by the Fc region of human immunoglobulin G (IgG) for one-step purification and the D10 sequence for targeting mineralizing tissue.^7^ Although ERT is a viable approach for the treatment of HPP, it requires repeated injection of large amounts of recombinant TNALP to obtain a curative effect because of the short half-life of TNALP-D_10_. Therefore, ERT has side effects (represented by injection site reactions) and is expensive, which are burdens for patients.^8^, ^9^

Gene therapy, which is expected to provide therapeutic effects through a single injection, represents an alternative treatment for HPP. Several studies using viral vectors (e.g., lentivirus or adeno-associated virus [AAV] vectors) as a gene therapeutic approach for lethal HPP have been performed to date.^10^, ^11^, ^12^, ^13^ Advantages of the AAV vector include the lack of diseases associated with a wild-type (WT) virus, the ability to transduce non-dividing cells, and the long-term expression of the delivered transgenes.^14^ AAV vector gene therapy shows long-term supplementation and easy accessibility and has been indicated to be safe in clinical trials with the first European Union-approved AAV type 1 vector (Glybera).^15^ We had previously reported that transduction of the TNALP-D_10_-expressing self-complementary AAV type 8 (scAAV8) vector into *Akp2*^*−/−*^ (TNALP-knockout) mice improved the skeletal bone defects in animals and prolonged their survival.^12^, ^13^ The AAV vector is suitable for *in vivo* gene delivery and has been widely used for vector-mediated ERTs for genetic diseases. We have shown that single-stranded (ss) AAV vector expression via the tissue-nonspecific CAG promoter (a hybrid promoter of the actin gene promoter and the cytomegalovirus immediate-early enhancer), can treat HPP in *Akp2*^*−/−*^ mice though a single injection.^12^ However, the potential problems of this approach include the risk of unexpected adverse effects from vector transduction into non-target tissues, including the gonads.^16^ As an alternative approach, the feasibility of scAAV8 vector-mediated TNALP-D_10_ gene therapy using a vector harboring the muscle-specific creatine kinase (MCK) promoter (scAAV8-MCK-TNALP-D_10_) delivered by intramuscular injection was considered, because the skeletal muscle is a practical target for gene therapy.

To investigate a more effective approach for HPP therapy, several experiments have been performed using *Akp2*^*−/−*^ mice. These mice mimic the same phenotype of human infantile HPP, since they are born with the normal mineralized skeleton, but then develop rickets and die within 3 weeks of birth. *Akp2*^*−/−*^ mice develop severe skeletal hypomineralization, apnea, and vitamin B6-responsive seizures.^17^, ^18^, ^19^ They also develop dental defects, including those of the cementum, dentin, alveolar bone, and enamel.^20^, ^21^, ^22^, ^23^, ^24^

Although several experiments have reported the therapeutic effects of ERT or gene therapy on dental defects in the lethal HPP mouse model, their improvement levels relative to normal tissue are not clear for defects of the cementum, dentin, or alveolar bone. Furthermore, the effect of gene therapy using the AAV vector for dental defects has not been fully investigated. The dentoalveolar area would be susceptible in HPP, as assumed by the existence of odonto-HPP. In addition, gene therapy is expected to prevent the exfoliation of teeth, thereby improving the quality of life of HPP patients by preventing mastication and retaining aesthetics. Therefore, in this study, we evaluated the therapeutic effects of scAAV8-MCK-TNALP-D_10_ on the mandibular bone and teeth of the HPP phenotype.

## Results

### Improved Mandibular Growth in scAAV8-MCK-TNALP-D_10_-Injected *Akp2*^*−/−*^ Mice

The scAAV8-MCK-TNALP-D_10_-treated *Akp2*^*−/−*^ mice (hereafter denoted TNALP-D_10_ mice) survived up to day 90, whereas the untreated *Akp2*^*−/−*^ mice died within 3 weeks. We first investigated the degree of mandibular growth and mineralization in TNALP-D_10_ mice by radiographic examinations.

**Figure 1:**
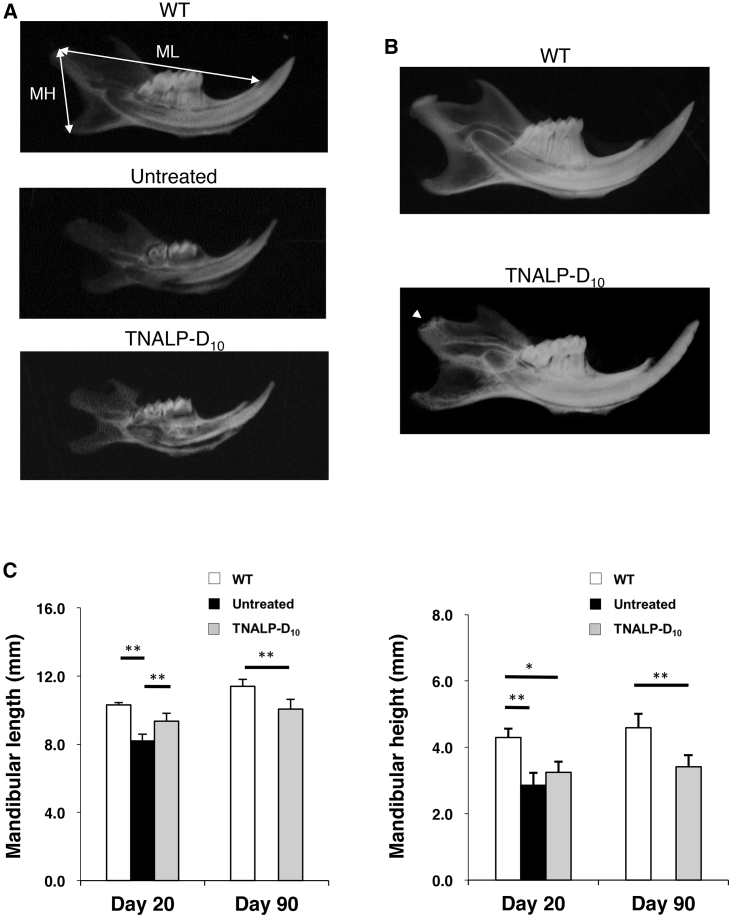
Radiographic Images and Linear Analysis of the Mandible (A) Radiographic images of the mandible at day 20 from wild-type mice (WT), untreated *Akp2*^*−/−*^ mice (Untreated), and TNALP-D_10_-treated *Akp2*^*−/−*^ mice (TNALP-D_10_) (n = 7). (B) Radiographic images of the mandible at day 90 from WT and TNALP-D_10_ mice (n = 7). The mandibular condyle of the TNALP-D_10_ mice was inferior in growth (arrowhead). (C) Linear analysis of the mandibular length (ML) and mandibular height (MH) indicated in (A). Data represent the means ± SD; *p < 0.05, **p < 0.01. TNALP-D_10_, tissue-nonspecific alkaline phosphatase with deca-aspartates at the C terminus.

At day 20, there was no distinction between the number of teeth and the structure of the mandible between the WT and *Akp2*^*−/−*^ mice (Figure 1A). The mandibular length of the TNALP-D_10_ mice was significantly longer than that of the untreated mice (Figure 1C; means ± SD, TNALP-D_10_ versus untreated, 955.3 ± 33.6 versus 758.8 ± 89.5 mm, *p* < 0.01). At day 90, the mandibular condyle of the TNALP-D_10_ mice was inferior in growth (Figure 1B). Both values of the mandibular length and mandibular height were significantly shorter in the TNALP-D_10_-treated mice than in the WT mice (Figure 1C; means ± SD, TNALP-D_10_ versus WT; mandibular length, 1,202.4 ± 60.8 versus 1,045.7 ± 29.1 mm, *p* < 0.01; mandibular height, 92.8 ± 3.2 versus 76.8 ± 12.9 mm, *p* < 0.01). These results suggested that mandibular growth in the TNALP knockout mice was improved by the scAAV8-MCK-TNALP-D_10_ injection, although the growth level was still under the normal level.

### Progressive Mineralization of the Molar Root in scAAV8-MCK-TNALP-D_10_-Treated Mice

**Figure 2:**
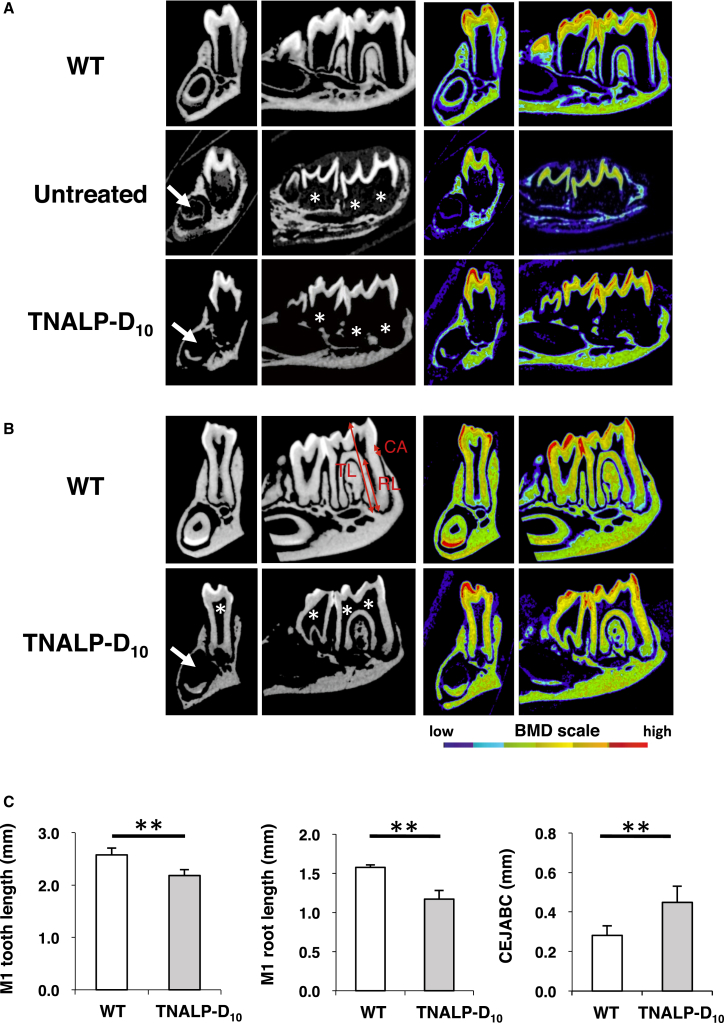
Micro-computed Tomography Grayscale and BMD Color Images and Linear Analysis of the Mandibular Molar Teeth Sections were cut in the coronal plane at the first molar (M1) distal root and sagittal plane. (A) Micro-computed tomography images from wild-type mice (WT), untreated *Akp2*^*−/−*^ mice (Untreated), and TNALP-D_10_-treated *Akp2*^*−/−*^ mice (TNALP-D_10_) at day 20 (n = 7). In the *Akp2*^*−/−*^ mice, the defects of the incisor teeth (arrows) and unsatisfied formation of the molar roots (asterisks) were identified. (B) Micro-computed tomography images from WT and TNALP-D_10_ mice at day 90 (n = 7). Expanded pulp chambers (asterisks) were observed in TNALP-D_10_ mice. (C) Linear analysis of the M1 tooth length in (B), the M1 root length, indicated as RL in (B), and the distance between the cemento-enamel junction and the alveolar bone crest (CEJABC), indicated as CA, in (B) at day 90. Data represent the means ± SD; **p < 0.01. TNALP-D_10_, tissue-nonspecific alkaline phosphatase with deca-aspartates at the C terminus.

We performed micro-computed tomography (micro-CT) scanning to investigate the association between scAAV8-MCK-TNALP-D_10_ treatment and hard tissue formation of the teeth and alveolar bone. At day 20, the molar crowns of the TNALP-D_10_ mice were observed, but not the molar roots, like those of the untreated mice (Figure 2A). At day 90, formation of the molar roots had progressed in the TNALP-D_10_ mice, whereas the pulp chambers were expanded compared with those of the WT mice (Figure 2B), implying the influence of dentin thinning. Furthermore, the tooth length and root length of the first molar (M1) were significantly shorter in the TNALP-D_10_ mice than in the WT mice (Figure 2C; means ± SD, TNALP-D_10_ versus WT; M1 tooth length, 2.60 ± 0.11 versus 2.18 ± 0.11 mm, *p* < 0.01; M1 root length, 1.58 ± 0.04 versus 1.17 ± 0.11 mm, *p* < 0.01). The incisor teeth were not always sufficiently mineralized in the TNALP-D_10_ and untreated mice (Figures 2A and 2B). These results suggest that tooth mineralization in TNALP-D_10_ mice had progressed with their growth.

### Improved Mineralization of the Alveolar Bone and Molar Teeth following TNALP-D_10_ Injection

Micro-CT analysis showed severe hypomineralization of the molar root and alveolar bone at day 20 in both the TNALP-D_10_ and untreated mice (Figure 2A). At day 90, mineralization of the alveolar bone had improved in the TNALP-D_10_ mice (Figure 2B). We next investigated the mineralization of the alveolar bone in greater detail by examining several micro architectural parameters, namely the bone mineral density (BMD; mg/cm^3^), bone volume fraction (BV/TV; %), trabecular thickness (Tb.Th; μm), trabecular number (Tb.N; /mm), and trabecular separation (Tb.Sp; μm).

**Figure 3:**
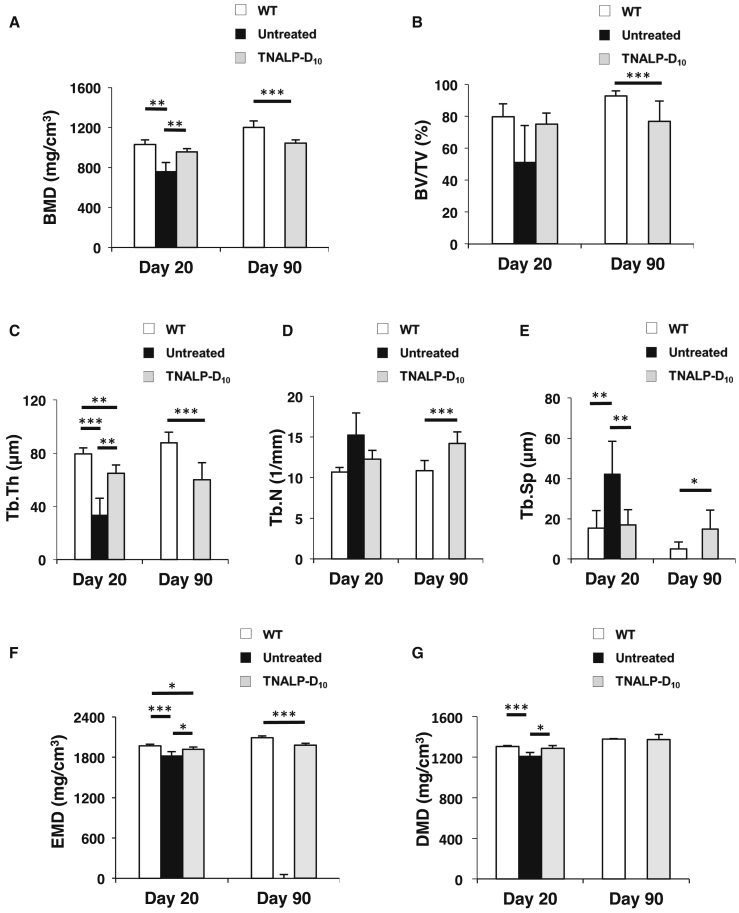
Morphometric Evaluation of the Alveolar Bone and Teeth Calculated at the Proximal Area (A) BMD, bone mineral density; (B) BV/TV, bone volume/tissue volume ratio; (C) Tb.Th, trabecular thickness; (D) Tb.N, trabecular number; (E) Tb.Sp, trabecular separation; (F) EMD, enamel mineral density; and (G) DMD; coronal dentin mineral density. The analysis was performed using wild-type mice (WT), untreated *Akp2*^*−/−*^ mice (Untreated), and TNALP-D_10_-treated *Akp2*^*−/−*^ mice (TNALP-D_10_) (n = 7). Data represent the means ± SD; *p < 0.05, **p < 0.01, ***p < 0.005. TNALP-D_10_, tissue-nonspecific alkaline phosphatase with deca-aspartates at the C terminus.

At day 20, the BMD and Tb.Th values were significantly higher in the TNALP-D_10_ mice than in the untreated mice (Figures 3A and 3C; means ± SD, TNALP-D_10_ versus untreated; BMD, 955.3 ± 33.6 versus 758.6 ± 88.5 mg/cm^3^; Tb.Th, 65.0 ± 6.0 versus 33.2 ± 12.8 μm; respectively, *p* < 0.01), and the Tb.Sp value of the TNALP-D_10_ mice was significantly lower than that of the untreated mice (Figure 3E; means ± SD, TNALP-D_10_ versus untreated; 16.9 ± 7.6 versus 42.3 ± 16.1 μm, *p* < 0.01). These results suggest that the structure and mineralization of the alveolar bone were improved by the scAAV8-MCK-TNALP-D_10_ injection. On the other hand, significant differences between the TNALP-D_10_ and WT mice were detected in all morphometric parameters at day 90 (Figures 3A–3E; means ± SD, TNALP-D_10_ versus WT; BMD, 1,045.7 ± 29.1 versus 1,202.4 ± 60.7 mg/cm^3^; BV/TV, 76.8 ± 12.9% versus 92.8 ± 3.2%; Tb.Th, 60.1 ± 12.8 versus 88.0 ± 7.7 μm; Tb.N, 14.2 ± 1.4 versus 10.9 ± 1.2 /mm; Tb.Sp, 14.9 ± 9.3 versus 4.9 ± 3.6 μm; respectively, *p* < 0.05). In addition, the distance between the cemento-enamel junction (CEJ) and the alveolar bone crest of the TNALP-D_10_ mice was significantly longer than that of the WT mice (Figure 2C; means ± SD, TNALP-D_10_ versus WT; 0.45 ± 0.08 versus 0.28 ± 0.05 μm, *p* < 0.01). These data suggest that the alveolar bone of the TNALP-D_10_ mice still showed hypomineralization and abnormal bone structure.

We also investigated the enamel mineral density (EMD; mg/cm^3^) and coronal dentin mineral density (DMD; mg/cm^3^) of M1. At day 20, EMD and DMD values were significantly higher in the TNALP-D_10_ mice than in the untreated mice (Figures 3F and 3G; means ± SD, TNALP-D_10_ versus untreated; EMD, 1,915.3 ± 36.4 versus 1,828.1 ± 54.9 mg/cm^3^; DMD, 1,286.0 ± 31.2 versus 1,208.4 ± 36.1 μm; respectively, *p* < 0.05). These results suggest that the mineralization of the enamel and coronal dentin were improved by the scAAV8-MCK-TNALP-D_10_ injection. On the other hand, significant differences between the TNALP-D_10_ and WT mice were still detected in EMD at day 90 (Figure 3F; means ± SD, TNALP-D_10_ versus WT; 2,090.9 ± 23.6 versus 1,978.8 ± 28.2 mg/cm^3^, *p* < 0.001). These data suggest that the enamel of the TNALP-D_10_ mice still showed hypomineralization, though it wasn’t shown in the coronal dentin.

### Dentoalveolar Formation in scAAV8-MCK-TNALP-D_10_-Treated Mice

**Figure 4:**
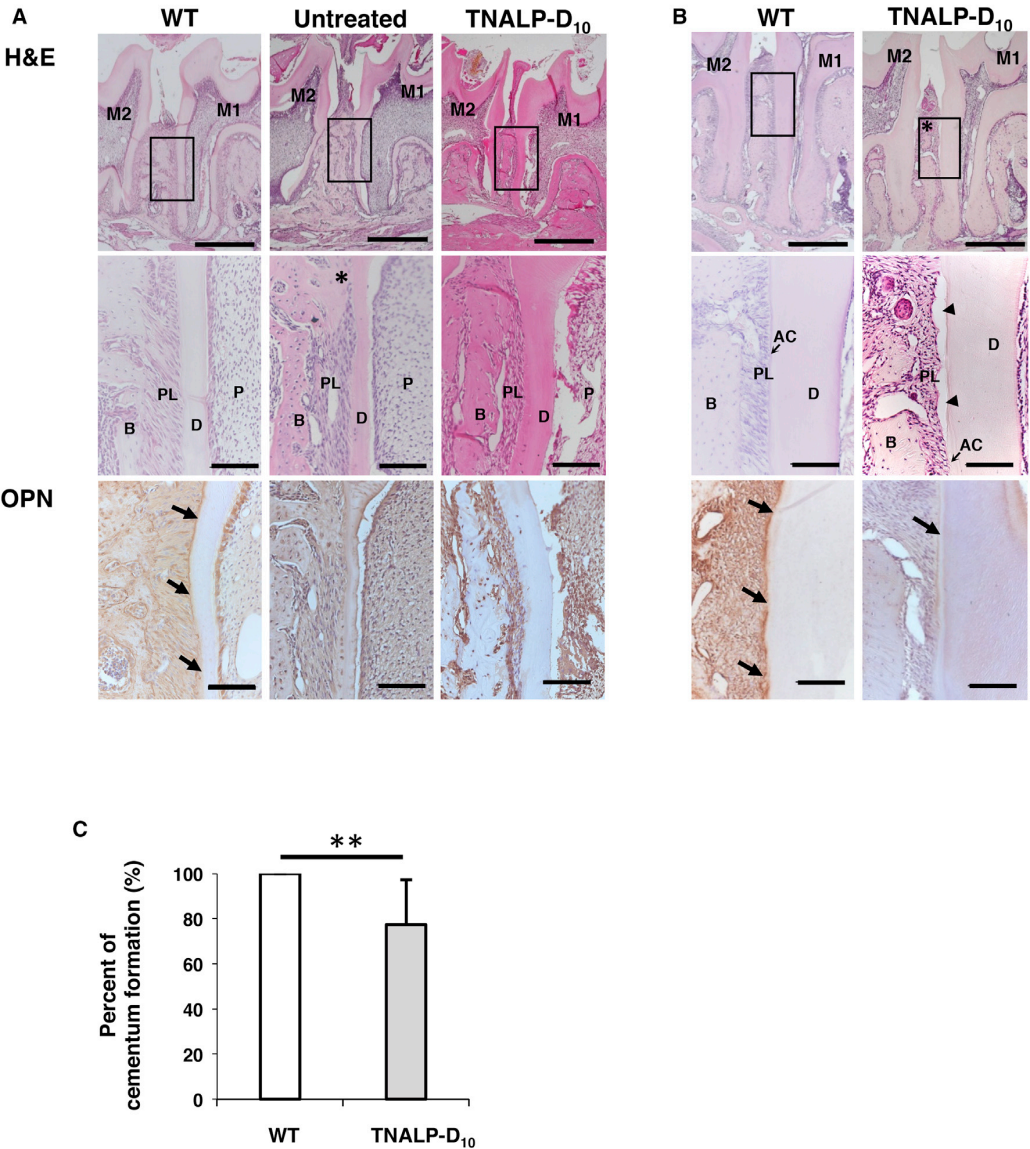
Histologic Analysis of Mandibular Molar Teeth (A) H&E staining of the molar teeth (upper panels, ×50; scale bars, 500 μm) and the first molar distal cervical region (middle panels, ×200; scale bars, 100 μm) and immunohistochemical staining with osteopontin at the first molar distal cervical region (lower panels, ×200; scale bars, 100 μm) of 20-day-old wild-type mice (WT, left panels), untreated *Akp2*^*−/−*^ mice (Untreated, center panels), and TNALP-D_10_-treated *Akp2*^*−/−*^ mice (TNALP-D_10_, right panels). Loss of the ligament width, suggesting the presence of tooth ankylosis (asterisk in the middle center panel), was observed in some of the *Akp2*^*−/−*^ mice. The fibroblasts in periodontal ligament were arranged regularly in WT mice, whereas the arrangement was irregular in the untreated and TNALP-D_10_ mice. Arrows in the lower right panel indicate osteopontin on the cementum layer. Osteopontin around the root surface was not confirmed in the untreated and TNALP-D_10_ mice. (B) H&E staining of molar teeth (upper panels, ×50; scale bars, 500 μm) and the first molar distal cervical region (middle panels, ×200; scale bars, 100 μm) and immunohistochemical staining with osteopontin at the first molar distal cervical region (lower panels, ×200; scale bar, 100 μm) of 90-day-old WT and TNALP-D_10_ mice. Decline of the alveolar bone crest (asterisk in the upper left panel) and defects of the cementum (arrowheads in the middle left panel) were observed in the TNALP-D_10_ mice. In addition, the localization of osteopontin around the root surface (arrows in the lower right panel) was ambiguous in the TNALP-D_10_ mice but clearly localized in the WT mice. (C) Percentage of cementum formation at the distal root of the first molar. Data represent the means ± SD. **p < 0.01. M1, first molar; M2, second molar; OPN, osteopontin; B, alveolar bone; PL, periodontal ligament; D, dentin; P, pulp; AC, acellular cementum; TNALP-D_10_, tissue-nonspecific alkaline phosphatase with deca-aspartates at the C terminus.

To further investigate the dentoalveolar area of the scAAV8-MCK-TNALP-D_10_-treated mice, histologic analysis was performed by H&E staining and immunohistochemical staining with osteopontin. At day 20, formation of the molar root and alveolar bone could be detected in the TNALP-D_10_ and untreated *Akp2*^*−/−*^ mice by histologic analysis (Figure 4A), although these formations were not detected by micro-CT analysis (Figure 2A). These findings suggest that formation of the molar root and alveolar bone had progressed in the *Akp2*^*−/−*^ mice as a result of the scAAV8-MCK-TNALP-D_10_ treatment, but mineralization of the structures had been delayed by the TNALP defect, and their improvement in the TNALP-D_10_ mice was still below the normal level. Loss of periodontal ligament width was seen in some of the untreated or TNALP-D_10_ mice, suggesting the presence of tooth ankylosis. The fibroblasts in periodontal ligament were arranged regularly in WT mice, whereas the arrangement was irregular in the untreated and TNALP-D_10_ mice. Immunohistochemical localization of osteopontin around the root surface was not confirmed in the TNALP-D_10_ mice.

At day 90, the alveolar bone crest of the TNALP-D_10_ mice was lower than that of the WT mice (Figure 4B). Partial hypoplasia of the acellular cementum localized on the cervical aspect of the root was observed in the TNALP-D_10_ mice (Figure 4B). Therefore, the rate of cementum formation in the treated mice was significantly lower than that in the WT mice (Figure 4C; means ± SD, TNALP-D_10_ versus WT, 77.4 ± 22.8% versus 100.0 ± 0%; *p* < 0.01). Immunohistochemical analysis of the localization of osteopontin around the root surface revealed ambiguous results for the TNALP-D_10_ mice, but clearly showed localization in the WT mice (Figure 4B). This suggested that improvement of cementum formation in the TNALP-D_10_ mice had remained at lower levels, and the structural combination of the teeth and alveolar bone was weaker than that in the WT mice.

In this study, hypomineralization or defect of the alveolar bone and cementum was identified more conspicuously around the interproximal area than around the proximal area, and therefore the histological analysis was focused in the interproximal area. On the other hand, morphological analysis of the alveolar bone was investigated in the proximal area because formation of the alveolar bone was hardly seen in the interproximal area at day 20 using micro-CT.

## Discussion

We had previously reported that scAAV8-MCK-TNALP-D_10_-mediated muscle transduction sustained plasma alkaline phosphatase (ALP) activity levels beyond 1.0 U/mL, which has been shown to prolong the survival of *Akp2*^*−/−*^ mice and provide them with partial skeletal improvement.^13^ In this study, we focused on the mandibular bone and teeth, which were apparently commonly affected in HPP in the same experiment system as our previous study,^13^ and we demonstrated that the same transduction enables improved growth and mineralization of the mandibular bone and teeth, although such improvements were still under the normal level. These results indicate that the plasma ALP activity levels (over 1.0 U/mL) were efficacious for the growth and mineralization of bone and teeth in *Akp2*^*−/−*^ mice. However, the ALP activity in the bone of the *Akp2*^*−/−*^ mice was still lower than the normal level after the scAAV8-MCK-TNALP-D_10_ treatment.^12^ It is difficult to transduce scAAV vector into the hard tissue directly, so we previously demonstrated that a single injection of recombinant scAAV8 vector expression via the tissue-nonspecific CAG promoter had an effective therapeutic benefit. However high-levels of ALP activity were detected in the nontargeted tissues, and the safety of systemic injection of viral vector has not been established.^12^, ^16^ In addition, we previously reported that dendritic cells (DCs) were activated by AAV8, suggesting that DCs play key roles in the immune response against AAV-mediated transduction.^25^ Therefore, we chose intramuscular injection to minimize the inflammatory reaction caused by AAV injection. In addition, we used the MCK promoter as a safer and more practical approach because the skeletal muscles are easily accessible and have a large capacity for protein production.^26^ TNALP was expressed specifically in the muscle by scAAV8-MCK-TNALP-D_10_-mediated muscle transduction, and then it went to circulation.^13^ High levels of ALP activity were restricted to the muscle (means ± SD, TNALP-D_10_ versus WT; 0.164 ± 0.084 versus 0.007 ± 0.003 U/mg, *p* < 0.01, at day 90), and ALP activity was not remarkable in the heart, lung, or liver using the MCK promoter, which was the same experiment system of this study. We also confirmed that plasma ALP activity levels of TNALP-D_10_ mice were increased more than 10-fold compared with that of WT mice (means ± SD, TNALP-D_10_ versus WT; 1.27 ± 0.69 versus 0.087 ± 0.025 U/mL, *p* < 0.001, at day 90), though bone ALP activity did not reach the level of WT mice.^13^ TNALP localizes on the plasma membrane of osteoblasts and hypertrophic chondrocytes via a GPI anchor and has a role in hydrolyzing PPi and releasing ionic inorganic phosphate (Pi) to create conditions that are conducive for mineralization.^27^ On the other hand, TNALP-D_10_ does not connect with the plasma membrane because it lacks the GPI anchor. Our previous study demonstrated that ALP activities in the bone of TNALP-D_10_ mice did not reach the level of WT mice in the same experiment system, even though the plasma ALP activities of TNALP-D_10_ mice were increased more than 10-fold over the dose in WT mice.^13^ Therefore, further enhancement of plasma ALP activity is necessary in HPP to increase the ALP activity in the bone.

The morphology of the mandibular condyle seemed to be insufficient in *Akp2*^*−/−*^ mice, likely due to the background of mandibular hypogrowth. Formation of the mandibular bone occurs through several ossification processes: the body of the mandible is formed by intramembranous ossification, whereas the mandibular condyle is formed by endochondral ossification. This latter ossification process also forms the long bone, vertebra and cranial base bones. Previous reports have shown that growth and mineralization defects of the long bone, vertebra, and cranial base were evident in *Akp2*^*−/−*^ mice and patients with HPP.^13^, ^28^, ^29^ From these findings, it is assumed that a similar defect would occur in the mandibular condyle of HPP patients. Abnormal chondrocyte arrangement was observed in the femur growth plate of *Akp2*^*−/−*^ mice, and, therefore, the same may be observed for the mandibular condyle.^19^ Although observations on the hard tissue of HPP have been reported for many bone regions, including the craniofacial bone, the effect of HPP on the mandibular bone is still unknown. In this study, mandibular growth was significantly improved by the scAAV8-MCK-TNALP-D_10_ treatment for HPP. A detailed characterization of the growth and mineralization of the individual parts of the mandible will be necessary to achieve further improvement of mandibular conditions.

Although premature tooth exfoliation occurs more frequently in the incisor of HPP patients, we focused our investigation on the molar teeth. The reason is that the incisor of rodents has specific structural and growth characteristics, whereas the molar of rodents has a similar structure to that of humans. The tooth is anchored by the periodontal fibers, which connect the cementum and the alveolar bone. Therefore, cementum defects are commonly the main risk of premature tooth exfoliation in patients with HPP. Other exfoliation risks are abnormalities of the alveolar bone and/or periodontal ligament, although the relationship between tooth exfoliation in HPP and their defects is still uncertain. By the way, abnormal dentoalveolar formations involving the cementum and alveolar bone were observed in the molar teeth of the severe infantile HPP mouse model.^20^, ^21^, ^23^ This suggested that cementum and alveolar bone defects could be a cause of tooth exfoliation in patients with lethal HPP. However, mineralization of the alveolar bone was improved by scAAV8-MCK-TNALP-D_10_, indicating that the risk of premature tooth exfoliation in HPP could be restricted by this treatment.

Therapeutic approaches for HPP have been reported by some groups. ERT or gene therapy using the lentiviral vector could improve dental defects in *Akp2*^*−/−*^ mice. *Ex vivo* therapy using bone marrow cells transduced with a TNALP-D_10_-expressing lentiviral vector partially improved cementum and alveolar bone defects at day 30 after birth.^30^ In this study, we were able to assess the mandibular bone and teeth at day 90 in the TNALP-D_10_ mice, because of their prolonged survival. It is still unclear whether the dentoalveolar condition is improved by scAAV8-MCK-TNALP-D_10_ treatment in the adult TNALP-D_10_ mouse. Nevertheless, we suggest that the safety of scAAV8-MCK-TNALP-D_10_ gene therapy gives it potential for not only prolonging survival and growth, but also for the formation of acellular cementum, dentin, and alveolar bone in patients with lethal HPP, although some of the risks of tooth exfoliation would remain. Furthermore, it was reported that HPP patients have a risk of exfoliation of the permanent teeth, as well as the deciduous teeth.^31^ Prosthodontic rehabilitations, such as bridges, dentures, or dental implants, are difficult in adult HPP patients who have lost their teeth, owing to their poor dentoalveolar condition. Therefore, we need further development of AAV-mediated gene therapy to recover the dentoalveolar condition of HPP completely. As one solution, the effectiveness of fetal gene therapy using the AAV9 vector for lethal HPP was reported, where formation of the human tooth germ was initiated in the fetal stage.^32^ Thus, as for fetal gene therapy, further improvement of the dentoalveolar area is expected.

Compared with untreated mice, mineralization of enamel and coronal dentin were improved in TNALP-D_10_ mice. However, obvious improvement of the tooth root was not observed in them. Defect of the enamel is associated with the risk of caries or tooth wear. Therefore, improvement of the enamel mineralization can be expected to protect the internal dentin and pulp from damage due to mechanical overload and exposure to the harsh chemical environment of the mouth.

Recombinant TNALP-D_10_-treated *Akp2*^*−/−*^ mice showed complete recovery of mineralization in all of the incisor tooth tissues, molar dentin, and surrounding alveolar bone after birth. This approach also improved HPP-related defects in the cementum, enamel, and dentin.^20^, ^22^, ^24^ Therefore, it is expected that the combination of scAAV8-MCK-TNALP-D_10_ gene therapy and ERT will have further therapeutic effects on the bones and teeth of HPP patients, thereby decreasing both the frequency of ERT administration and its inherent burdens on these patients.

In conclusion, our results suggest that the mandibular bone and teeth of *Akp2*^*−/−*^ mice were effectively treated by recombinant scAAV8-mediated muscle TNALP-D_10_ transduction. For the next stage of the study, the adequate dose of TNALP for complete bone cure and teeth safety without adverse events will be established through improved transfection strategies or vector dose with immune modulation.

## Materials and Methods

### Gene Transduction of Animals

All animal experiments were performed in accordance with the guidelines approved by the Nippon Medical School Animal Ethics Committee. *Akp2*^*+/+*^ WT mice and TNALP knockout *Akp2*^*−/−*^ mice were obtained by mating *Akp2*^*+/−*^ mice with a mixed genetic background of 129/J and C57BL/6J, which were generated by the Millán laboratory (La Jolla, CA). The mice were fed a rodent diet supplemented with pyridoxine (vitamin B6) to suppress seizures.^33^ Genotyping was performed by the PCR as described in previous reports.^13^ For the TNALP-D_10_ expression, scAAV8-MCK-TNALP-D_10_ (2.5 × 10^12^ vector genomes [vg]/body weight diluted with PBS to a total volume of 15 μL) was injected into the bilateral quadriceps femoris muscle of neonatal *Akp2*^*−/−*^ mice on day 1. All treated and untreated mice were sacrificed at 20 and 90 days of age and perfused with 15 mL of PBS containing heparin (10 U/mL) followed by 15 mL of PBS. Age-matched WT mice were used as the controls. Each group used 7 mice for the evaluations.

### X-Ray Analysis

Radiographs of the mandibles were taken with a μFX-1000 system (Fujifilm, Tokyo, Japan) using an energy level of 25 kV. The radiographs were exposed for 30 s and imaged with a Typhoon FLA-7000 scanner (Fujifilm). Linear analysis of the mandibular length (the distance between the mandibular condyle and the lingual alveolar bone crest of the incisor) and height (the distance between the mandibular condyle and the mandibular angle) was conducted.

### Micro-CT Analysis

Micro-CT scans of single mandibular hemi-sections were performed at the molar level using an R-mCT scanner (Rigaku, Tokyo, Japan) with the following imaging conditions: matrix size, 512 × 512; tube voltage, 70 kV; tube current, 120 μA; magnification, × 10; slice width, 20 μm; and voxel size, 20 × 20 × 20 μm. Coronal and sagittal planes were chosen for comparison. The total length (the distance between the mesiolingual cusp and the apical foramen) and root length (the distance below the furcation of the total length) of M1 were measured. The distance between the CEJ and the alveolar bone crest was also measured. Each linear measurement was performed in the sagittal plane (parallel to tooth axis of M1) using i-view software.

### Morphometric Evaluation of the Alveolar Bone and Teeth

The digital image obtained was processed to reconstruct three-dimensional (3D) images using reconstruction software (TRI/3D-BON; Ratoc, Tokyo, Japan) to assess alveolar bone and molar formation. The parameters analyzed the alveolar bone by TRI/3D-BON were the BMD (mg/cm^3^), BV/TV (%), Tb.Th (μm), Tb.N (/mm), and Tb.Sp (μm). The region of interest was evaluated in the alveolar bone around the proximal area of M1. We also analyzed the EMD (mg/cm^3^) and the coronal DMD (mg/cm^3^) of M1 by TRI/3D-BON.

### Tissue Preparation and Histologic Analysis

Mandibular hemi-sections obtained at 20 and 90 days after birth were fixed in 4% paraformaldehyde-PBS for 3 days and then transferred to 70% ethanol for storage at 4°C. They were then decalcified in 7.5% EDTA containing 7% sucrose for 2 weeks at 4°C with gentle agitation, with the EDTA solution changed every 2 days, and then embedded in paraffin. Sagittal sections (2 μm) around the distal root of M1 were mounted on silane-coated slides and subjected to H&E staining and immunohistochemical analysis. The percentage of cementum formation was investigated by measuring the length of cementum defective region in the distal surface of the root, from the CEJ to the root apex. For immunohistochemical analysis of osteopontin, a marker of acellular cementum, a rabbit polyclonal anti-mouse osteopontin antibody (1:500, Immuno-Biological Laboratories, Gunma, Japan) was used as the primary antibody.^34^ The bound primary antibody was detected with a Dako EnVision+ System using a horseradish peroxidase labeled anti-rabbit secondary antibody (Dako, Tpokyo, Japan) and visualized with diaminobenzidine as the substrate. The sections were then counterstained with hematoxylin and observed under a light microscope (UPM Axiophot2, Carl Zeiss, Oberkochen, Germany).

### Statistical Analysis

Statistical significance, set at *p* < 0.05, was evaluated using the Kruskal-Wallis test with the post hoc Mann-Whitney U test.

## Author Contributions

R.I. collected and analyzed the data and drafted the article. A.N.-T., Y.N.-K., A.W., T.M., and T.O. designed the study and revised the article critically. T.S. approved the final article version to be published.

## Conflicts of Interest

The authors have no conflicts of interest to declare.
